# Starters vs. non-starters differences in vertical jump force-time metrics in female professional volleyball players

**DOI:** 10.3389/fspor.2024.1389001

**Published:** 2024-03-25

**Authors:** Damjana V. Cabarkapa, Dimitrije Cabarkapa, Andrew C. Fry

**Affiliations:** Jayhawk Athletic Performance Laboratory—Wu Tsai Human Performance Alliance, Department of Health, Sport and Exercise Sciences, University of Kansas, Lawrence, KS, United States

**Keywords:** sport, force, power, performance, athlete monitoring, team sports

## Abstract

As one of the fundamental volleyball skills, countermovement vertical jump (CMJ) has been commonly implemented in the applied sports setting as a non-invasive and time-efficient assessment of athletes' lower-body neuromuscular function. The purpose of the present study was to examine the differences in CMJ characteristics between starters and non-starters within a cohort of professional female volleyball players. Nineteen athletes competing in one of the top European leagues (i.e., SuperLeague) volunteered to participate in the present investigation. Following the completion of a warm-up protocol, each athlete performed three maximal-effort CMJs with no arm swing while standing on a uni-axial force plate system sampling at 1,000 Hz. The following force-time metrics were used for performance analysis purposes: braking phase duration and impulse, eccentric and concentric duration, mean and peak force and power, contraction time, jump height, and reactive strength index-modified. Mann–Whitney *U* and independent *t*-tests revealed no statistically significant differences (*p *> 0.05) during both eccentric and concentric phases of CMJ between the players included in the starting lineup (*n* = 9) and their substitutions (*n* = 10), with the effect sizes being small to moderate in magnitude (*g *= 0.053–0.683). While further research is warranted on this topic, these results suggest that securing a position in a starting lineup at the professional level of volleyball play may be more contingent on the player's ability to proficiently execute sport-specific skills (e.g., blocking, attacking), rather than the performance on the CMJ assessment, considering that the observed values for both groups fall within the desired ranges for this specific population of athletes.

## 1 Introduction

Volleyball is one of the most popular international team sports. It is characterized by frequent high-intensity and change of direction movements (e.g., blocking, attacking) with short periods of rest (1–7). Based on the on-court competitive demands, it is well-known that volleyball players need to possess adequate levels of speed, agility, aerobic capacity, and upper-body and lower-body muscular strength and power to attain peak performance (3, 8).

As one of the fundamental volleyball-specific skills, the countermovement vertical jump (CMJ) has been commonly implemented in the applied sports setting for non-invasive and time-efficient monitoring of athletes' lower-body neuromuscular function (2, 9, 10). Although various technologies have been used to assess CMJ performance (e.g., accelerometers, force plates, three-dimensional motion capture systems), the force plate system remains a “gold standard” or criterion measure due to its ability to provide the practitioners with not only the outcome metrics (e.g., jump height) but an in-depth analysis of the mechanisms underpinning this specific movement (11, 12).

Previous scientific literature has been primarily focused on examining the physical and physiological attributes of volleyball players, as well as their on-court playing performance (9, 13, 14). Significant differences have been observed between different sexes (male vs. female) (14), playing positions (libero vs. outside hitter) (9, 14), competitive levels (professional vs. collegiate) (13, 15), and players' readiness to compete status (starters vs. non-starters) (16). For example, it has been found that national team volleyball players (i.e., top-tier players in one country) tend to have superior CMJ performance in comparison to both professional and collegiate athletes (15). Similar findings have been observed by Sattler et al. (14), showing that vertical jump height was considerably greater in both male and female volleyball players competing at the First Division in all jump performance assessments (i.e., CMJ, squat jump, block jump, and attack jump) when compared to the Second Division players. The same group of authors has found that male setters tend to display significantly lower CMJ height when compared to the receivers, while no significant position-specific differences were observed in female volleyball athletes (14). Moreover, an investigation done by Fry et al. (16) revealed the presence of considerable differences between athletes included in the starting lineup and their substitutions at the National Collegiate American Association (NCAA) Division-I level of play. Specifically, the authors indicated that starters on the team appeared to be faster, more flexible, and stronger compared to the non-starters (16).

Based on the aforementioned research reports, there is still a lack of scientific research focused on examining the performance characteristics specific to elite female volleyball players, especially considering the availability and usage of the force plate systems for neuromuscular performance assessment in applied sports settings. Therefore, the purpose of the present study was to identify whether differences in CMJ force-time metrics during both eccentric and concentric phases of the movement exist between starters and non-starters at the professional level of volleyball competition.

## 2 Materials and methods

### 2.1 Participants

Nineteen female volleyball players (age = 21.6 ± 2.8 years; height = 181.5 ± 7.0 cm; body mass = 74.2 ± 8.3 kg) competing in one of the top European leagues (i.e., SuperLeague) volunteered to participate in this study (*n* = 3 setters; *n* = 4 middle blockers; *n* = 6 outside hitters, *n* = 4 opposite hitters, *n* = 2 liberos). The athletes were classified as starters (*n* = 9) if they were a part of the starting lineup in >75% of games played during a regular season span and the rest were classified as non-starters (*n* = 10). All athletes were free of musculoskeletal injuries that could possibly impair their volleyball performance and were previously cleared by their respective sports medicine staff to participate in the team activities. The testing procedures were approved by the University's Institutional Review Board and all athletes signed the informed consent document.

### 2.2 Testing procedures

After completion of a 15-min standardized warm-up routine consisting of dynamic stretching exercises (e.g., lateral lunges, high-knees, A-skips, butt kicks) administered by the team's strength and conditioning coach, all athletes performed three maximal-effort CMJs with no arm swing (i.e., hands on the hips throughout the entire movement), while standing on a uni-axial force plate system (ForceDecks Max, VALD Performance, Brisbane, Australia) sampling at 1,000 Hz. During the testing procedures, all athletes were verbally encouraged to give their maximum effort during each jump and focus on pushing the ground as explosively as possible (17). Also, a 10–15 s rest interval was provided between each jump trial to minimize the possible influence of fatigue (2, 18).

### 2.3 Dependent variables

Based on the previously published scientific literature (2, 15, 18–21), the following force-time metrics of interest were examined: braking phase duration and impulse (excluding participant's body mass), eccentric and concentric duration, peak velocity, mean and peak force, and mean and peak power. In addition, contraction time, vertical jump height (i.e., impulse-momentum relationship), countermovement depth, and reactive strength index (RSI)-modified (i.e., jump height divided by contraction time) were also measured. A detailed description of the CMJ force-time variables can be found in the previous research reports (2, 15, 18, 19, 21) as well as in the VALD manual (https://valdperformance.com/forcedecks/).

### 2.4 Statistical analysis

Descriptive statistics, means (standard deviations) or median (interquartile range) were calculated for each dependent variable examined in the present study. Prior to the analysis, the Shapiro-Wilk test and Levene's test were used to examine if the assumption of normality and homogeneity of the data were violated, respectively. If the assumption was violated, the Mann–Whitney *U* test was selected to examine the differences in CMJ force-time metrics between the starters and non-starters on the professional female volleyball teams. If the assumption was not violated, the independent samples t-test was used for analysis. In addition, Hedge's g (*n* < 20) was calculated to investigate the magnitude of the differences between the groups. Based on Hedges (22), *g *= 0.2 was considered a small effect size, *g *= 0.5 moderate effect size, and *g *> 0.8 large effect size. Lastly, statistical significance was set *a priori* to *p *< 0.05 and all statistical analyses were performed in the SPSS (Version 26.0; IBM Corp., Armonk, NY, USA).

## 3 Results

No statistically significant differences were observed between starters and non-starters in regard to age (22.6 ± 2.9 years vs. 20.8 ± 2.6 years), height (182.7 ± 4.8 cm vs. 180.5 ± 8.7 cm), and body mass (72.6 ± 4.7 kg vs. 74.5 ± 10.7 kg), respectively. In addition, between-group differences for any of the CMJ force-time metrics examined in the present study were not observed. The effect sizes were small to moderate in magnitude for all performance metrics within both eccentric and concentric phases of the CMJ (*g *= 0.053–0.683), except for countermovement depth, which was large in magnitude (*g *= 0.804). See Table 1 for detailed results.

**Table 1:** Descriptive statistics, mean (standard deviation) or median (interquartile range), statistically significant differences, and Hedge's *g* effect sizes of CMJ force-time metrics between starters and non-starters.

Variable [unit]	Starters	Non-starters	*p*-value	Hedge's *g*
Eccentric phase				
Braking phase duration [s]	0.32 (0.16)	0.30 (0.09)	0.243	0.156
Eccentric braking impulse [Ns]^a^	43.2 (9.7)	45.1 (12.0)	0.700	0.173
Eccentric duration [s]	0.50 (0.08)	0.54 (0.11)	0.453	0.412
Eccentric peak velocity [m/s]	−1.23 (0.23)	−1.09 (0.18)	0.162	0.683
Eccentric peak force [N]	1619.6 (244.5)	1779.4 (288.5)	0.213	0.595
Eccentric mean force [N]	712.8 (46.1)	742.0 (104.9)	0.452	0.353
Eccentric peak power [W]	1298.2 (488.2)	1195.5 (407.7)	0.624	0.229
Eccentric mean power [W]	447.7 (60.6)	396.4 (105.2)	0.217	0.589
Concentric phase				
Concentric impulse [Ns]	174.4 (17.4)	179.8 (23.3)	0.579	0.260
Concentric duration [s]	0.29 (0.42)	0.27 (0.34)	0.210	0.053
Concentric peak velocity [m/s]	2.52 (0.20)	2.50 (0.12)	0.839	0.123
Concentric peak force [N]	1649.4 (217.9)	1784.9 (271.4)	0.250	0.547
Concentric mean force [N]	1330.9 (152.3)	1431.0 (217.4)	0.266	0.528
Concentric peak power [W]	3231.8 (469.4)	3321.7 (490.4)	0.689	0.187
Concentric mean power [W]	1766.0 (302.3)	1909.3 (279.4)	0.298	0.494
Other				
Contraction time [s]	0.79 (0.11)	0.81 (0.14)	0.833	0.158
Vertical jump height [cm]	29.7 (5.3)	29.1 (3.1)	0.794	0.140
RSI-modified [ratio]^a^	0.42 (0.12)	0.38 (0.07)	0.720	0.413
Countermovement depth [cm]	−31.1 (2.6)	−28.0 (4.7)	0.097	0.804

^a^ Non-normally distributed variables; RSI-modified, reactive strength index modified.

## 4 Discussion

To the best of our knowledge, this is the first study focused on examining differences in CMJ force-time metrics between starters and non-starters within an elite cohort of professional female volleyball players. The results revealed that both groups tend to display similar lower-body neuromuscular performance characteristics as measured during CMJ. No significant differences were observed in any CMJ force-time metrics of interest between starters and non-starters, including anthropometric characteristics, with a majority of effect sizes being small to moderate in magnitude.

The findings of the present study are in line with some of the previously published scientific literature focused on examining differences in physiological and anthropometric characteristics between starters and non-starters in various team sports such as basketball and soccer (23–25). For example, Yamaguchi et al. (24) compared the physical attributes of starters and non-starters within a cohort of elite-level professional female soccer players with similar morphological characteristics (e.g., height, age, body mass, muscle mass, body fat percentage). While non-starters displayed inferior performance in the field tests (i.e., 10 m sprint and 5 × 10 m shuttle run) than starters, both groups attained similar results on all other laboratory-based assessments such as CMJ and 40-m sprint tests (24). Similar observations were made by Risso et al. (23) when studying a cohort of NCAA Division-I female soccer players where similar physiological capacities were observed regardless of whether the athlete was a part of the starting lineup or not. Specifically, starters and non-starters demonstrated similar performance in the vertical jump (0.50 m vs. 0.50 m), standing broad jump (1.97 m vs. 1.92 m), and 30 m sprint assessments (4.69 s vs. 4.79 s) (23). Moreover, the results of the present study are in direct agreement with a recently published research report that found no differences in the same CMJ force-time metrics between starters and non-starters on a professional level of men's basketball competition (i.e., impulse, duration, peak velocity, and mean and peak force and power) as well as the outcome (e.g., jump height) and strategy metrics (e.g., countermovement depth) (25). Thus, based on the aforementioned findings, we can conclude that securing a position in a starting lineup cannot be solely determined by differences in lower-body neuromuscular performance characteristics from a CMJ test. While further research is warranted on this topic, we can assume that the player's ability to proficiently execute sport-specific skills (e.g., blocking, attacking), including the coach's evaluation of the player's performance capabilities, may have a greater impact on differentiating starters from non-starters than solely performing CMJ assessment (26).

On the other hand, Fry et al. (16) compared the physical and performance characteristics of starters and non-starters in NCAA Division-I female volleyball players and found that players who started the game were faster (∼0.28 s during the 36.6 m sprint test), stronger (i.e., greater maximal strength for bench press, military press, and hang power clean exercises), and more flexible (∼8.9 cm during sit and reach test) than the non-starters on the team. Moreover, Gabbett et al. (27) indicated that starters seemed to be taller, heavier, and have a better change of direction speed in elite and/or sub-elite junior rugby players when compared to the non-starters. Although the differences between the aforementioned groups might be present in different sports (e.g., rugby vs. volleyball) and levels of competition (i.e., collegiate vs. professional), it should be noted that the majority of the physical and performance variables (e.g., one-repetition maximum, jump height) could be improved with an appropriate and comprehensive strength and conditioning training program (16). In addition, while not reaching the level of statistical significance, it is interesting to note that the countermovement depth was slightly greater in starters than in non-starters (*g *= 0.804). This observation may imply a possible presence of differences in the CMJ movement strategy or technique used to attain a certain level of jump performance (2). However, this assumption falls beyond the scope of the present study, and further research is warranted to obtain a comprehensive insight into biomechanical differences in CMJ movement patterns.

While providing a better insight into neuromuscular performance characteristics of professional female volleyball players, this study is not without limitations. The sample of the athletes participating in the study could have been larger, including sampling frequency (e.g., 2–3 testing time points across a competitive season). Also, future research needs to examine if the observed findings remain applicable to other competitive levels (e.g., collegiate or youth athletes) as well as if they are sex-specific (e.g., male vs. female).

In conclusion, the findings of the present study indicate that CMJ assessment might not be a useful tool when determining contributing factors to success at the professional level of women's volleyball competitions. Both starters and non-starters demonstrated similar performance on all force-time metrics of interest during both eccentric and concentric phases of the CMJ movement, with small to moderate effect size differences. Overall, these findings imply that other parameters such as the player's ability to proficiently execute sport-specific skills (e.g., blocking, attacking) may have a greater impact on differentiating players included in the starting lineup from their substitutions at the professional level of play.

## Data Availability

The raw data supporting the conclusions of this article will be made available by the authors, without undue reservation.
